# Prevalence of Healed Plaque and Factors Influencing Its Characteristics Under Optical Coherence Tomography in Patients With Coronary Artery Disease: A Systematic Review, Meta-Analysis, and Meta-Regression

**DOI:** 10.3389/fcvm.2021.761208

**Published:** 2021-11-22

**Authors:** Xunxun Feng, Yang Liu, Jiaqi Yang, Guangyao Zhai, Yujie Zhou, Qianyun Guo

**Affiliations:** Beijing Key Laboratory of Precision Medicine of Coronary Atherosclerotic Disease, Department of Cardiology, Clinical Center for Coronary Heart Disease, Beijing Institute of Heart Lung and Blood Vessel Disease, Beijing Anzhen Hospital, Capital Medical University, Beijing, China

**Keywords:** prevalence, healed plaque, characteristics, optical coherence tomography, coronary artery disease, systematic review, meta-analysis, meta-regression

## Abstract

**Aim:** The purpose of this study was to determine the prevalence of healed plaque and its characteristics under optical coherence tomography (OCT) through a formal systematic review, meta-analysis, and meta-regression.

**Methods and Results:** Thirteen studies were selected from MEDLINE, EMBASE, Cochrane, and online databases. The overall incidence of healed plaques was 40% (95% CI: 39–42), with 37% (95% CI: 35–39) in patients with acute coronary syndrome (ACS) and with 46% (95% CI: 43–49) in patients with stable angina pectoris (SAP). The incidence of healed plaque among culprit plaques (48%, 95% CI: 46–50) was nearly two times higher than that among non-culprit plaques (24%, 95% CI: 21–27). The incidence of thin cap fibroatheroma (TCFA), plaque rupture, microvessel, macrophage accumulation, and calcification was significantly higher in the healed plaque group. Meta-regression revealed an association between smoking (*P* = 0.033) and healed plaque rupture. Gender (*P* = 0.047) was independently associated with macrophage accumulation, and mean low-density lipoprotein cholesterol (LDL-C) was independently associated with microvessel.

**Conclusions:** In summary, with a total incidence of 40%, the incidence of healed plaques under OCT was higher in SAP than in ACS, and higher in culprit plaques than in non-culprit plaques. Higher incidence of TCFA, plaque rupture, microvessel, macrophage accumulation, and calcification was found in the healed-plaque group. Smoking, gender, and mean LDL-C level were associated with healed-plaque characteristics.

## Introduction

Healed plaque is a type of coronary atherosclerotic plaque that is characterized by infiltration with inflammatory cells, endothelial cells, and smooth muscle cells, and its formation may be associated with a stable lesion-contained thrombus. The newly synthesized type III collagen in the early healing stage, which is rich with proteoglycan and loosely aligned, is eventually replaced by type I collagen in the late healing stage. Therefore, most of the thrombotic substances in different stages have a layered structure after plaque rupture. Thus, the most prominent feature of healed plaques is a layered structure of organized thrombus and/or collagen (1–3).

In terms of pathophysiology of the healed plaque, previous autopsy studies have shown a high incidence of healed plaques, ~61–73% in the culprit and non-culprit plaques (4). In addition, in an 18-month follow-up study using angioscopy to clarify the healing process of disrupted culprit plaques in patients with myocardial infarction, it was detected that the healing process of plaques showed the decrease in thrombogenicity and color grade, which were aggravated particularly in patients with diabetes mellitus and hyperlipidemia (5). Moreover, in non-culprit lesions, plaque healing and disruption may be a pan-coronary evolvement in acute myocardial infarction patients with the decreasing in thrombogenicity and yellow color grade during angioscopic observations among disrupted and actively thrombogenic yellow plaques (6). In addition, based on the importance of angiogenesis for plaque healing, Brezinski et al. showed that longitudinal angiogenesis presented long necrotic cores and failed to approach some areas in the intimal cap. As a result of impaired healing mechanisms for acute coronary syndrome (ACS), they thought that these areas were at risk for thinning, rupturing, or eroding (7).

Optical coherence tomography (OCT) is an intravascular imaging technique allowing a detailed view of the coronary artery wall structure. OCT can identify macrophages in atherosclerotic plaques, and its high resolution can detect thin cap fibroatheroma (TCFA), plaque rupture, and macrophage accumulation in most of the layered plaques (8, 9). Healed plaques are recognized by different optical features on different layers. These features reflect the acute events that have previously undergone the healing process. Thanks to its recent development and application, OCT has been proven to be a useful tool for the detection of healed plaques.

Healing of an atherosclerotic plaque is a dynamic process that occurs after plaque rupture, which could prevent thrombus, promote plaque renovation, and restore vascular integrity. However, the clinical significance of plaque healing remains a controversial issue. Plaque healing may help atherosclerotic patients avoid ACS, but it can lead to chronic coronary syndrome (10). In addition, healed plaques proved to be the predictor of rapid plaque progression as well as fatty plaques and TCFA, and a new layer was found in more than half of the rapidly progressing lesions during clinical study follow-up (11). Therefore, healed plaques play an important role in patients with coronary artery disease (CAD), and efficient identification of healed plaques by OCT provides a certain reference value for clinical practice.

We conducted a systematic review, meta-analysis, and meta-regression to evaluate the prevalence of healed plaques and their characteristics under OCT observation.

## Methods

### Protocol Registration and Standards

We carried out this study in accordance with the preferred reporting items for systematic reviews and meta-analyses (PRISMA) guidelines. The protocol of this meta-analysis was registered at PROSPERO, International Prospective Register of Systematic Reviews [CRD42020186412], and this conformed with the meta-analysis of observational studies in epidemiology guidelines (12) and preferred reporting items for systematic review and meta-analysis (13).

### Data Sources and Search Strategy

Three independent researchers (FXX, LY, and YJQ) searched MEDLINE, EMBASE, and Cochrane databases to identify potentially relevant studies from the inception of databases to Sep 20, 2021. The incorporating keywords including “healed,” “layered,” “healing,” and “healings” were searched and combined with headings from medical subject and Emtree including “Atherosclerotic Plaque,” “Optical Coherence Tomography,” and “Wound Healing” or “Healing” (Supplementary Tables 1–3).

### Study Selection

Three independent researchers (FXX, ZGY, and GQY) discussed the retrieved studies after reading their own title and/or abstract, with differences solved with consensus. The inclusion criteria were as follows: (1) *in vivo* OCT of coronary arteries conducted in clinical patients; (2) human studies including OCT examination and evaluating the characteristics of coronary plaques; (3) studies considering factors influencing healed plaque's morphology. The exclusion criteria were as follows: (1) duplicate reporting, conference abstract, or review; (2) studies that mentioned healed plaque but did not subgroup the data into healed plaque group and non-healed plaque group; (3) studies that could not explain factors influencing healed-plaque morphology.

### Types of Studies and Definition

Longitudinal, cross-sectional, and interventional studies reporting baseline data to assess characteristics of healed plaques were considered. Moreover, studies had to have been peer-reviewed and published in English to be included, and the assessment of the study quality was based on the Newcastle-Ottawa scale, as shown in Supplementary Table 4. Culprit plaques were identified by coronary angiography, electrocardiographic changes, left ventricular wall motion abnormalities, or echocardiography. In patients with multiple stenoses, plaques showing the most severe stenosis on angiography or OCT or with acute thrombosis were thought to be the culprits (14–16). In the meta regression, the incidence rates of various characteristics of healed plaque were the primary end points. Clinical factors influencing the on healed-plaque characteristics were tested using meta-regression analysis in the overall population and in different clinical subsets of patients such as ACS and stable angina pectoris (SAP) (17).

### Data Extraction and Quality Assessment

Two independent researchers (FXX and GQY) reviewed the titles and abstracts of all studies independently. The full texts of all potentially qualifying articles were assessed. To extract relevant information from 13 included studies, a standardized form was used. Two independent researchers (FXX and GQY) evaluated the risk of bias for the finally included 13 studies in accordance with the Cochrane Handbook for Systematic Reviews of Interventions. Disagreements were resolved by discussion among the researchers until they reached accordance.

### Statistical Analysis

Continuous variables were shown as mean ± standard deviation (SD). Values for categorical variables were listed as odds ratio (ORs) with 95% confidence interval (CI). The meta-analysis was implemented using the fixed-effects model when the *I*^2^ value was <50%. Otherwise, the random effects model was used. Original data were first converted by the double arcsine method to ensure their compliance with the normal distribution. The inverse variance method was used to calculate the pooled incidence of healed plaque and 95% CIs. Subgroup meta-analyses were conducted for categorical variables [classification of CAD (ACS v. SAP) and type of plaques (culprit vs. non-culprit)] (18, 19). For the outcomes of all data with statistical heterogeneity from moderate to high (*I*^2^ ≥ 50%), subgroup analyses were performed to investigate potential moderators of the effect. As previously reported (20), meta-regression was performed to reveal the potential sources of heterogeneity using random-effects models with the restricted maximum likelihood estimation and the adjustment of Knapp/Hartung for estimation of standard errors of the estimated coefficients, which were conducted for continuous variables when at least four studies reported on a given instrument [including demographics: age (mean years), gender (%male); laboratory examination: low-density lipoprotein cholesterol (LDL-C; mean); risk factors of CAD: diabetes mellitus (%), hypertension (%), hyperlipidemia (%), smoking (%), and statin therapy (%)]. The publication bias of the healed plaque prevalence and characteristics was judged by the funnel plot and Egger's test (21). *P*-value (two-tailed) < 0.05 was considered statistically significant. All data were analyzed using Stata/MP version 15 (StataCorp, College Station, Texas) and Review Manager 5.3.

## Results

A total of 369 potential studies were screened. Among them, 13 studies were identified for inclusion and further evaluation, and then two were excluded because they did not distinguish between healed and non-healed plaques. The complete process of identification and exclusion of studies is described in detail in the PRISMA flowchart (Supplementary Figure 1). A total of 3,141 patients were included in 13 studies, covering the period from 2018 to 2021 (Tables 1, 2). Further details of the coronary angiography and characteristics of OCT in each study are shown in Table 3.

**Table 1:** Characteristics of included studies.

**References**	**Location**	**Study design**	**No. of Patients with variety**	**Primary endpoint**	**Mortality reported**	**Follow-up time**
Shimokado et al. (22)	Japan	Retrospective	60; SAP	–	–	–
Fracassi et al. (8)	America	Retrospective	376; ACS	Cardiac death, acute myocardial infarction, ischemia-driven revascularization, and rehospitalization	Yes	1 year
Okamoto et al. (23)	Japan	Retrospective	166; SAP	–	–	–
Russo et al. (24)	America	Retrospective	349; ACS	–	–	–
Wang et al. (16)	China	Retrospective	204; ACS+SAP	Death from any cause, myocardial infarction, and target lesion revascularization	Yes	1 year
Araki et al. (25)	America	Retrospective	313; ACS+SAP	–	–	–
Kurihara et al. (26)	America	Retrospective	193; ACS+SAP	Cardiac death, myocardial infarction, and target lesion revascularization	Yes	329 ± 90 days
Kurihara et al. (27)	America	Retrospective	265; ACS+SAP	Cardiac death, ACS or revascularization	Yes	2 years
Russo et al. (28)	America	Prospective	163; SAP	–	–	–
Usui et al. (15)	Japan	Retrospective	538; SAP+ACS	Cardiac death, myocardial infarction, or ischemia-driven revascularization	Yes	3 years
Dai et al. (14)	China	Prospective	325; ACS	Cardiac death, recurrent myocardial infarction, ischemia-driven revascularization, or rehospitalization	Yes	1 year
Li et al. (29)	China	Prospective	156; ACS	–	–	–
Kimura et al. (30)	Japan	Retrospective	33; SAP	New-onset unstable angina pectoris, non-fatal myocardial infarction, target vessel revascularization, or cardiac death	Yes	474 days

*ACS, acute coronary syndrome; SAP, stable angina pectoris*.

**Table 2:** Baseline characteristics of included studies.

**Author**	**Male, %**	**DM, %**	**HT, %**	**Smoking, %**	**Previous PCI, %**	**Dyslipidemia, %**	**BMI**	**Age, y**	**TC, mg/dL**	**TG, mg/dL**	**LDL-C mg/dL**	**HDL-C mg/dL**
Shimokado et al.	80	48	92	55	48	80	UK	68 ± 10	UK	159 ± 83	103 ± 29	42 ± 11
Fracassi et al.	76	27	51	55	12	36	24 ± 6	58 ± 12	181 ± 44	138 ± 67	116 ± 39	45 ± 11
Okamoto et al.	73	43	80	51	46	74	24 ± 4	69 ± 12	165 ± 32	128 ± 66	98 ± 31	47 ± 13
Russo et al.	76	27	51	56	11	34	25 ± 3	57 ± 11	178 ± 42	138 ± 77	116 ± 40	45 ± 11
Wang et al.	81	28	64	34	27	67	26 ± 5	59 ± 11	179 ± 46	145 ± 79	102 ± 35	46 ± 20
Araki et al.	77	30	64	27	31	70	25 ± 4	61 ± 11	177 ± 44	161 ± 66	104 ± 38	45 ± 13
Kurihara et al.-1	78	36	64	26	UK	68	UK	62 ± 11	183 ± 46	161 ± 116	109 ± 39	44 ± 13
Kurihara et al.-2	80	28	63	28	UK	70	UK	60 ± 11	172 ± 44	169 ± 131	99 ± 36	43 ± 12
Russo et al.	79	27	69	19	40	71	25 ± 4	64 ± 10	157 ± 38	102 ± 65	89 ± 29	48 ± 16
Usui et al.	83	37	67	35	23	58	UK	67 ± 10	UK	UK	106 ± 34	46 ± 12
Dai et al.	74	21	40	51	UK	51	UK	56 ± 11	184 ± 41	117 ± 65	112 ± 37	54 ± 14
Li et al.	81	30	55	74	UK	82	26 ± 3	57 ± 11	UK	175 ± 42	113 ± 33	41 ± 9
Kimura et al.	82	61	52	73	UK	70	UK	68 ± 8	149 ± 82	UK	87 ± 35	47 ± 11

*BMI, body mass index; DM, diabetes mellitus; HT, hypertension; PCI, percutaneous coronary intervention; TC, total cholesterol; TG, triglycerides; LDL-C, low-density lipoprotein cholesterol; HDL-C, high-density lipoprotein cholesterol; UK, unknown*.

**Table 3:** Angiographic features and OCT-derived plaque characteristics.

**References**	**Angiographic features**				**Plaque Characteristics Analyzed**									
	**Culprit vessel**			**Multivessel disease, %**	**Qualitative analysis**						**Quantitative analysis**			
	**LAD, %**	**RCA, %**	**LCX, %**		**TCFA**	**Plaque rupture**	**Thrombus**	**Microvessel**	**Macrophages**	**Calcification**	**MLA**	**RLA**	**AS**	**Lesion length**
Shimokado et al. (22)	60	20	20	82	N	N	N	Y	Y	Y	Y	Y	Y	Y
Fracassi et al. (8)	54	35	11	44	Y	Y	Y	Y	Y	Y	Y	Y	Y	N
Okamoto et al. (23)	55	24	16	67	Y	Y	N	Y	Y	Y	Y	N	N	N
Russo et al. (24)	77	58	47	27	Y	Y	N	Y	Y	Y	Y	Y	Y	Y
Wang et al. (16)	53	30	17	51	Y	Y	Y	Y	N	Y	Y	Y	Y	Y
Araki et al. (25)	57	27	16	53	Y	Y	Y	Y	Y	Y	Y	Y	Y	N
Kurihara et al. (26)	60	25	16	UK	N	N	Y	N	Y	Y	Y	Y	Y	N
Kurihara et al. (27)	56	28	16	UK	N	N	N	N	Y	N	Y	Y	Y	N
Russo et al. (28)	61	20	18	54	Y	N	Y	Y	Y	Y	Y	Y	Y	Y
Usui et al. (15)	46	37	13	UK	Y	N	N	Y	Y	Y	Y	N	N	N
Dai et al. (14)	–	UK	UK	UK	Y	N	Y	Y	Y	Y	Y	Y	Y	Y
Li et al. (29)	–	UK	UK	UK	Y	Y	Y	Y	Y	Y	Y	N	N	N
Kimura et al. (30)	28	56	17	UK	Y	Y	N	Y	Y	Y	Y	Y	Y	N

*LAD, left anterior descending artery; RCA, right coronary artery; LCX, left circumflex artery; TCFA, thin-cap fibroatheroma; MLA, minimal lumen area; RLA, reference lumen area; AS, area stenosis; OCT, optical coherence tomography; N, No; Y, yes; UK, unknown*.

### Prevalence of Healed Plaque

**Figure 1:**
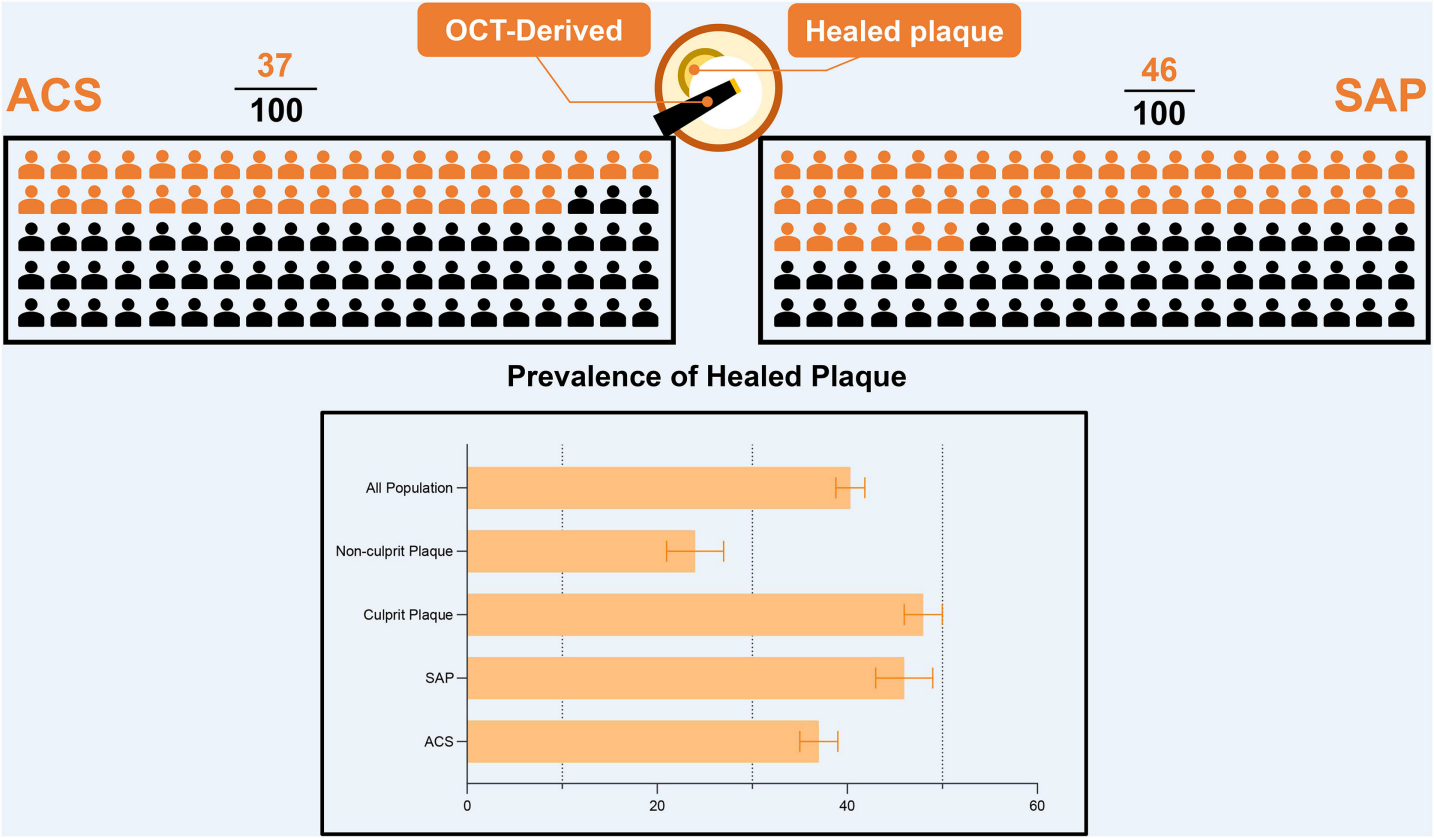
The prevalence of healed plaque in different population. OCT, optical coherence tomography; ACS, acute coronary syndrome; SAP, stable angina pectoris.

To analyze the prevalence of healed plaques, we performed a single-arm meta-analysis. In the general population, the incidence of healed plaques was 40% (95% CI: 39–42). Stratified by the manifestations of disease, the incidence of healed plaques was 37% (95% CI: 35–39) in the patients with ACS and 46% (95% CI: 43–49) in patients with SAP. Moreover, at the level of plaques, the incidence of healed plaques among culprit plaques (48%, 95% CI: 46–50) was higher than that among non-culprit plaques (24%, 95% CI: 21–27) (Figure 1). Heterogeneity among studies that evaluated healed plaques was generally extensive (*I*^2^ > 95%). On the one hand, the heterogeneity may have been driven by the variable estimation of healed-plaque morphology in the included studies and specific characteristics that introduced a degree of heterogeneity into the analysis. The specific definitions of each characteristic in healed plaque in every study are listed in Supplementary Table 5. On the other hand, in the culprit lesion intraplaque hemorrhage, the performance of thrombus aspiration prior to OCT imaging might have altered the underlying healed plaque morphology.

### Morphology of Healed Plaques

A total of 13 studies reported the morphological differences between healed plaque and non-healed plaque. Potential sources of heterogeneity were explored by subgroup analysis through traditional classification, including the type of CAD (ACS vs. SAP) and the type of plaques (culprit vs. non-culprit) from the included studies.

**Figure 2:**
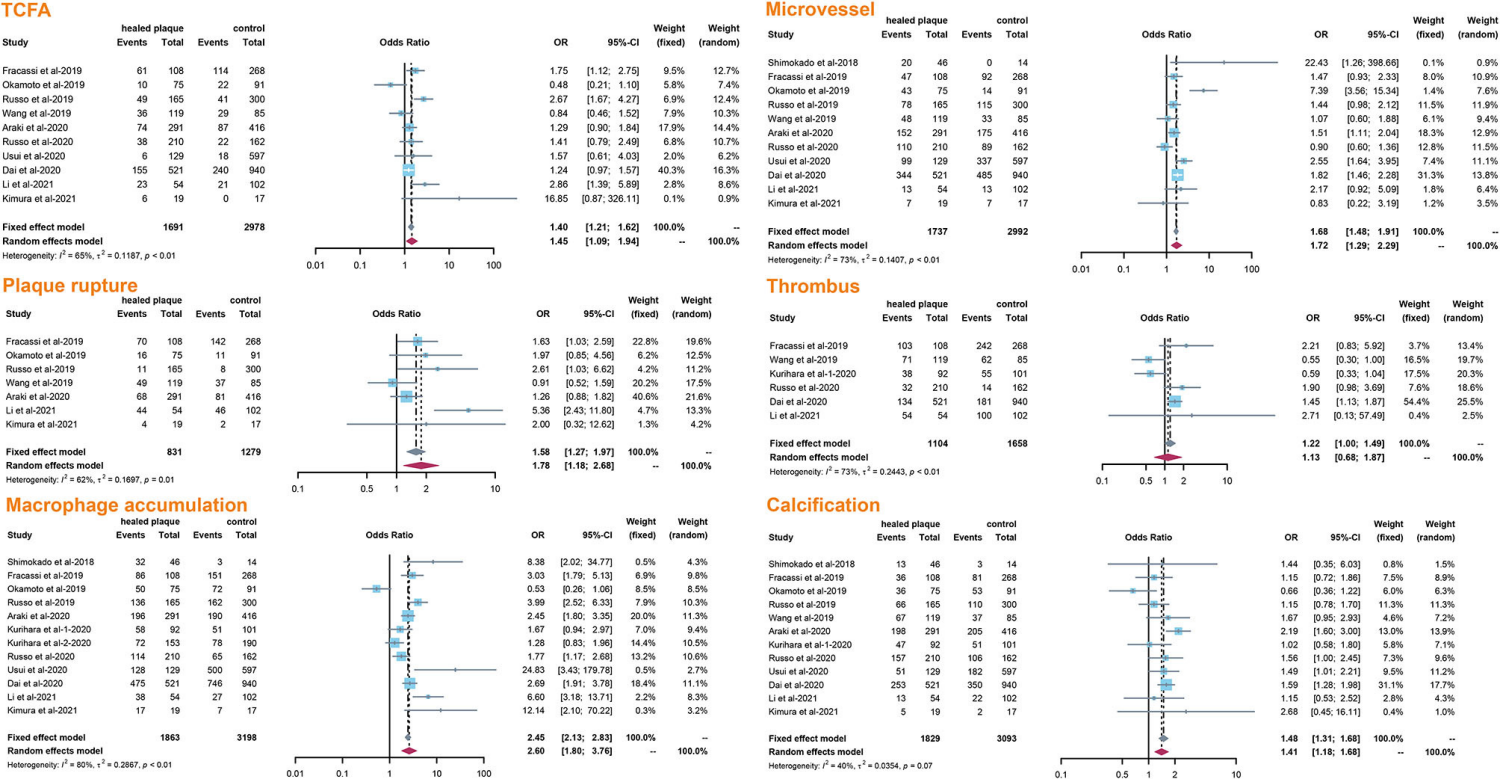
Summary of qualitative characteristics of healed plaques in the total population. TCFA, thin-cap fibroatheroma.

**Figure 3:**
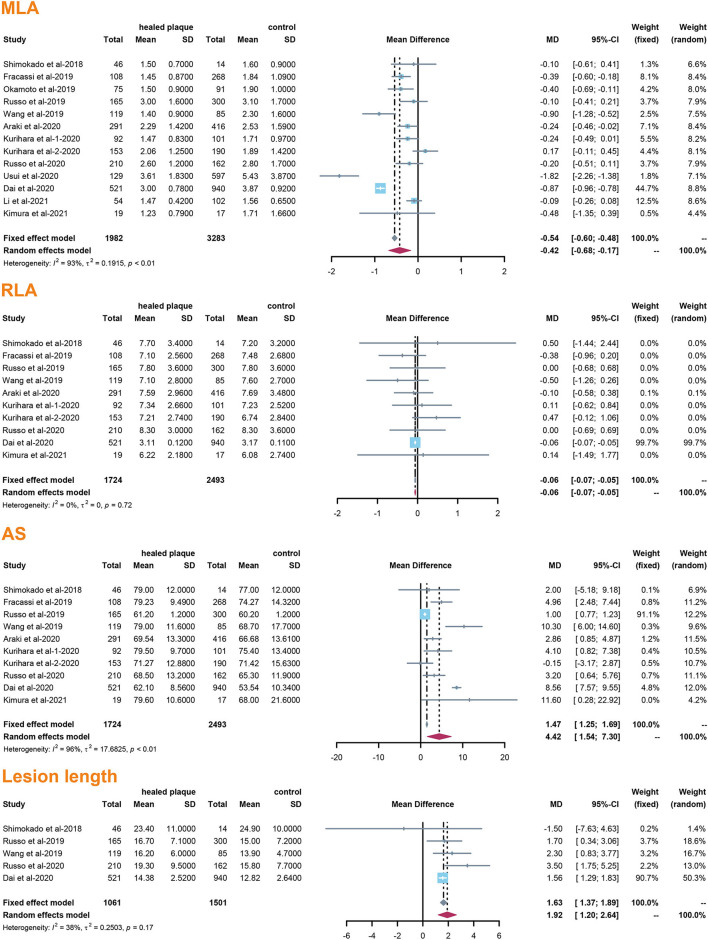
Summary of quantitative characteristics of healed plaques in the total population. MLA, minimal lumen area; RLA, reference lumen area; AS, area stenosis.

The incidence of TCFA, plaque rupture, microvessel, macrophage accumulation, and calcification was higher in the healed plaque group (Figure 2), whereas minimal lumen area (MLA) and reference lumen area (RLA) were smaller. Meanwhile, the area stenosis (AS) and lesion length were higher than those in the non-healed plaque group (Figure 3). According to the subgroup analysis of culprit and non-culprit plaques, the incidence of macrophage accumulation (*P* < 0.05) was generally higher in the healed plaques, whereas the microvessel and calcification were higher only in culprit group. MLA was lower in healed plaques, but AS was higher than that in non-healed plaques only in the culprit group. Lesion length was also greater in healed plaques only in the culprit group. In the ACS and SAP subgroups, the incidence of TCFA was higher in healed plaques. MLA was lower in healed plaques, while AS showed the opposite trend (Supplementary Figures 2–5).

### Meta Regression

**Figure 4:**
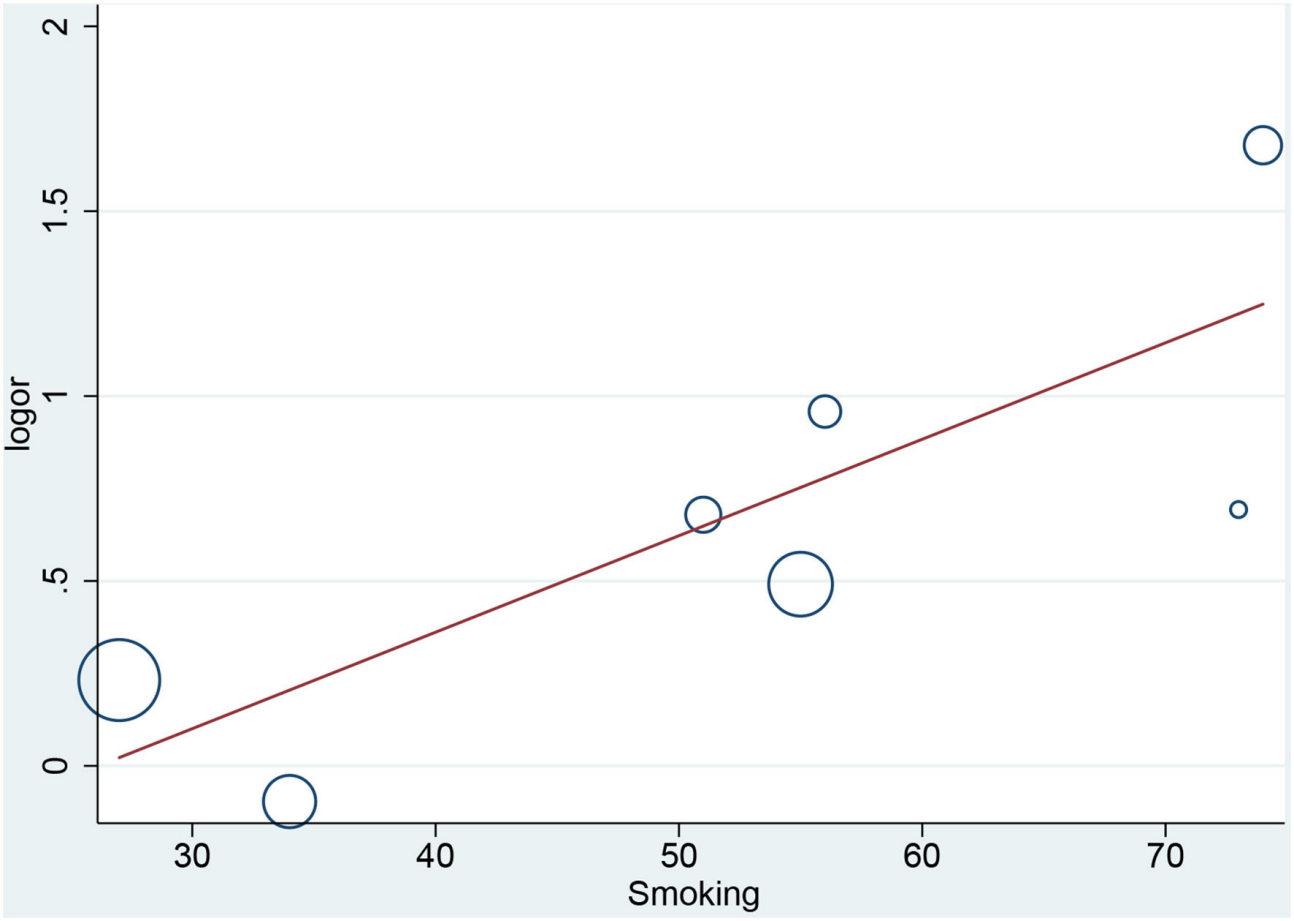
Meta-regression for smoking as an influence factor of plaque rupture in overall population.

**Figure 5:**
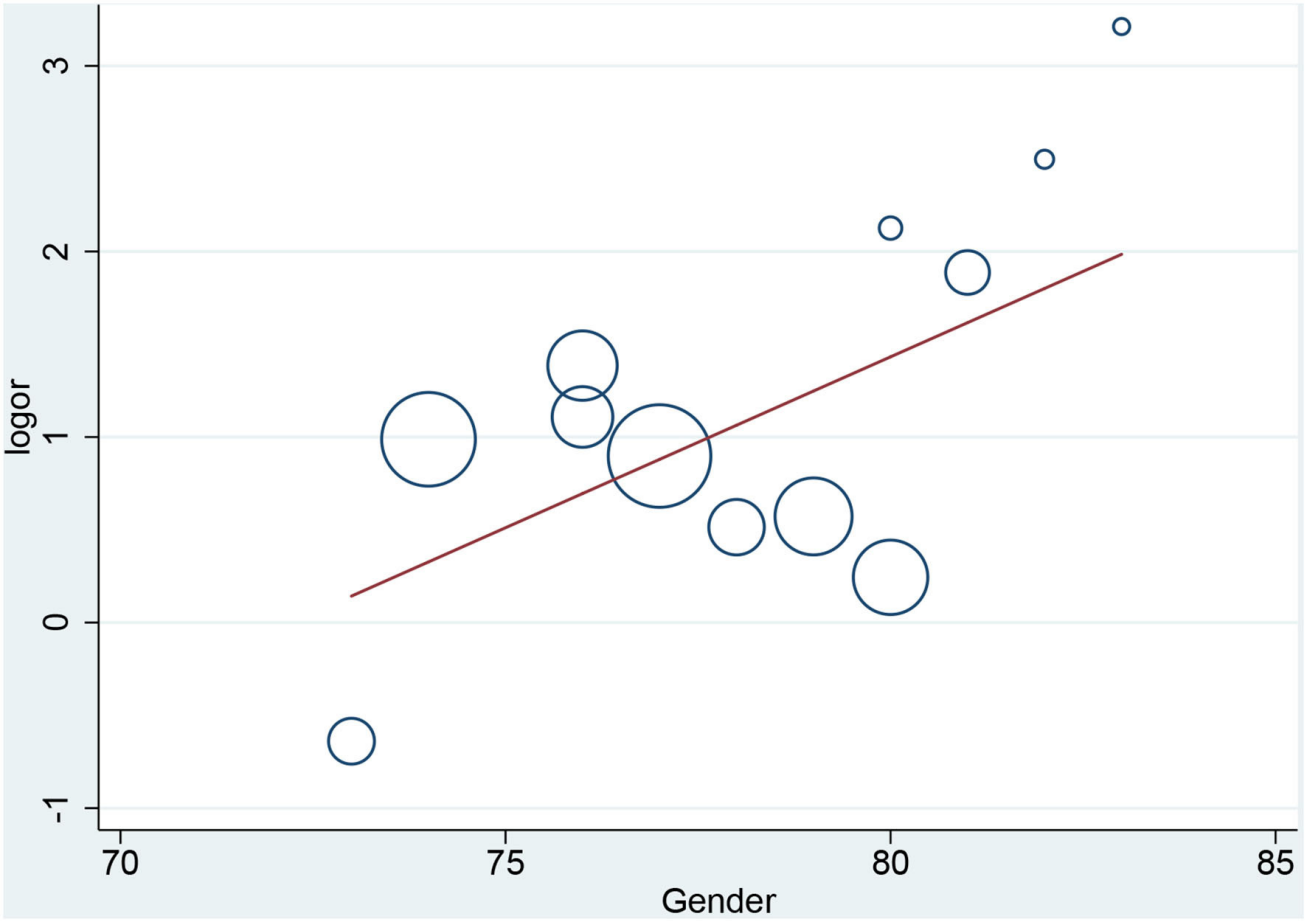
Meta-regression for gender as an influence factor of macrophage accumulation in overall population.

In the meta-regression, we did not find the potential source of heterogeneity in the culprit or non-culprit data. Regarding the characteristics of healed plaque, we observed that in the entire population smoking was associated with heterogeneity in healed-plaque rupture (*P* = 0.033), and gender (%male) was associated with heterogeneity in macrophage accumulation (*P* = 0.047) (Table 4, Figures 4, 5). Regarding the microvessel, the meta-regression analysis showed that the difference in the mean level of LDL-C was associated with heterogeneity among SAP patients (*P* = 0.034) (Table 5). However, the calcification, TCFA, and thrombus were not affected by the clinical factors (*P* > 0.05).

**Table 4:** Meta-regression of influence factors for plaque rupture and macrophage accumulation in healed plaque among overall population.

	**Plaque rupture**				**Macrophage accumulation**			
	**Point estimate**	**Lower limit**	**Upper limit**	***P*** **-value**	**Point estimate**	**Lower limit**	**Upper limit**	***P*** **-value**
Age	−0.024	−0.157	0.109	0.660	−0.016	−0.158	0.125	0.802
Male	0.011	−0.213	0.234	0.908	0.184	0.003	0.366	0.047
Hypertension	−0.017	−0.082	0.048	0.525	−0.015	−0.062	0.031	0.482
Diabetes	0.007	−0.067	0.080	0.827	0.021	−0.044	0.086	0.486
Hyperlipidemia	0.004	−0.032	0.041	0.782	−0.006	−0.046	0.034	0.739
Smoking	0.026	0.003	0.049	0.033	0.022	−0.008	0.053	0.133
TCFA	−0.979	−7.571	5.613	0.718	−1.441	−9.349	6.466	0.679
LDL-C	0.046	−0.338	0.126	0.199	0.023	−0.041	0.088	0.440
Statin therapy	–	–	–	–	0.992	0.979	1.004	0.159

*LDL-C, low-density lipoprotein cholesterol*.

**Table 5:** Meta-regression of influence factors for micovessel in healed plaque among SAP patients.

	**Point estimate**	**Lower limit**	**Upper limit**	***P*** **-value**
Age	0.369	−1.085	1.823	0.389
Male	−0.207	−1.088	0.674	0.419
Hypertension	0.086	−0.088	0.259	0.168
Diabetes	0.009	−0.307	0.325	0.910
Hyperlipidemia	0.466	−0.116	1.048	0.075
Smoking	0.012	−0.181	0.206	0.808
LDL-C	0.227	0.043	0.411	0.034

*LDL-C, low-density lipoprotein cholesterol*.

### Publication Bias

**Figure 6:**
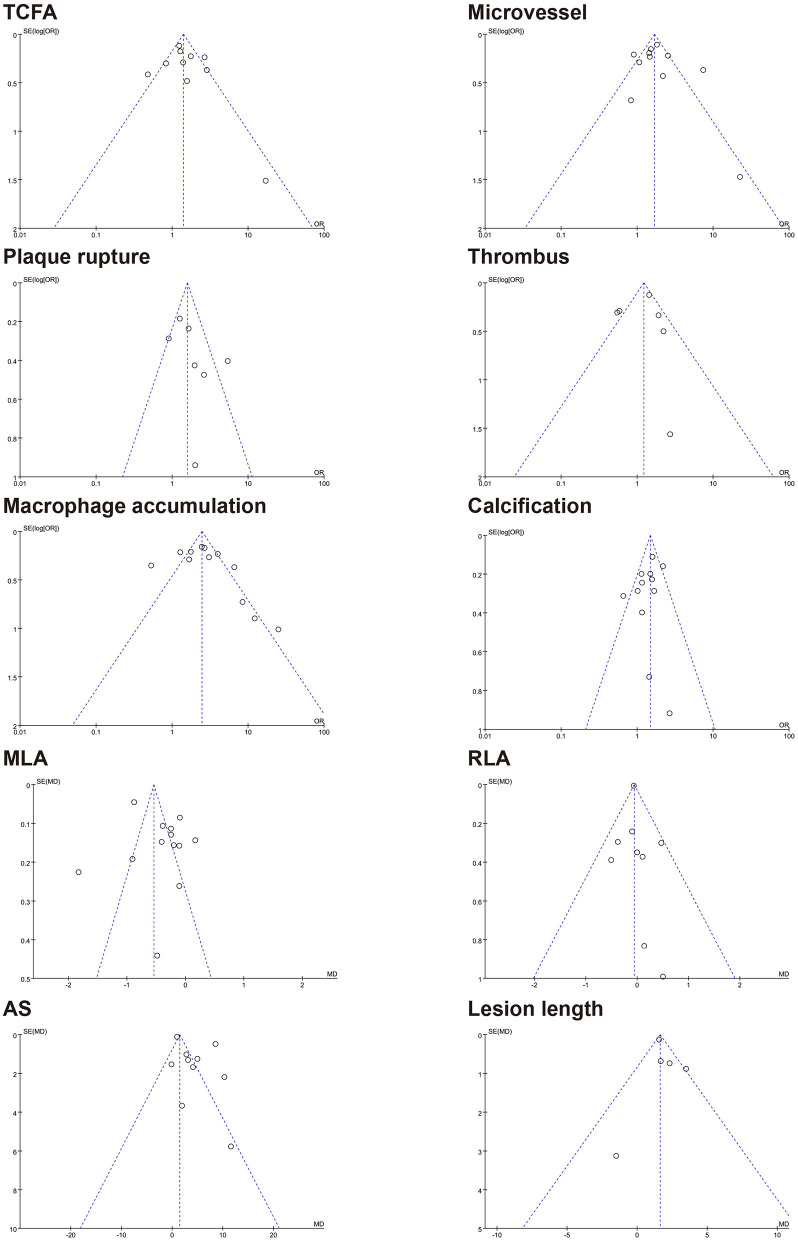
Funnel plot for characteristics of healed plaque. TCFA, thin-cap fibroatheroma; MLA, minimal lumen area; RLA, reference lumen area; AS, area stenosis.

The results of the funnel plot for characteristics of healed plaque are shown in Figure 6, and the results of Egger test for prevalence of healed plaque are shown in Supplementary Table 6. In addition, Egger test checking for the existence of publication bias, as described in earlier studies, was performed for every characteristic of the healed plaque (Supplementary Table 7). There was no publication bias in the characteristics of healed plaque according to the funnel plot, and Egger's test did not show any publication bias in the single-arm meta-analysis.

## Discussion

To our knowledge, this is the first meta-analysis to evaluate OCT-based prevalence, describe the morphology, and explore the influencing factors of healed plaques. Our main findings were as follows: (1) The incidence of healed plaques in the overall population was 40%, with a higher incidence in SAP subgroup and at culprit sites. (2) Higher vascular fragility was reflected in higher incidence of TCFA and macrophage accumulation in healed plaques; the burden of healed plaques was heavier in patients with culprit plaques and ACS, as reflected in higher AS and longer lesion length. (3) Traditional cardiovascular risk factors including smoking, gender, and levels of LDL-C were associated with the characteristics of healed plaques.

According to our analysis, the incidence of healed plaques was different between ACS and SAP subgroups. While Shimokado et al. reported that OCT detected healed plaques in 77% of SAP patients' target lesions (22), Okamoto et al. observed layered heterogeneous plaques in 36.6% of SAP patients with pathogenic lesions (23). In contrast, Fracassi et al. found that one-third of the lesions in ACS patients had multilayer structures with complicated angiography (8). According to our results, there was a higher incidence of healed plaques in SAP patients (46%) than in ACS patients (37%). Additionally, our results indicated that the prevalence of healed plaques was higher in culprit plaques (48%) than in non-culprit plaques (24%). As previously described, although non-culprit lesions associated with culprit events are more likely to approach 70% or more of plaque burden, and patients with non-culprit plaque rupture have a high-risk atherosclerosis phenotype of the whole coronary artery (31, 32), healed culprit plaque in patients has a more layered and a more lipid plaque phenotype with more characteristics of plaque vulnerability (24, 25, 28). However, based on the previous studies mentioned above, more OCT-based studies are needed to clarify the characteristics of healed plaques combined with non-culprit plaques with or without the pursuant culprit lesions.

Cracked plaques are characterized by a thin fibrous cap, a large lipid-filled necrosis core, and persistent inflammation (33). The thickness of fibrous cap is an important morphological feature to distinguish a ruptured plaque (34); indeed, it is universally acknowledged that plaque rupture occurs on a background of TCFA, displaying more features of plaque vulnerability, which is the most common mechanism of intracavitary thrombosis in coronary arteries (29, 30, 35, 36). In our study, the incidence of TCFA in healed plaques was significantly higher only in the ACS group. Therefore, while TCFA in healed plaques may not be strongly associated with plaque type, it seems to be closely related to acute events such as ACS. However, the heterogeneity could not be explained by TCFA either in subgroup analysis or in meta-regression. Interestingly, smoking might be associated with heterogeneity in healed plaque rupture. We supposed that, as a common cardiovascular risk factor, smoking might influence the stability of healed plaque and then lead to crack.

Macrophage accumulation was measured in 92.3% of the included studies, and it showed significant differences between healed plaque and non-healed plaque in the whole population, SAP group, and in culprit plaques. There is certain evidence that macrophages are a driving force in all stages of atherogenesis, from the earliest lesions to the formation of complex plaques (37); moreover, the available evidence suggests that increased multifocal and focal macrophage density highly correlates with the severity of CAD symptoms (38). The infiltration of healed plaques by macrophages might indicate the level of inflammatory response, suggesting that there might be a higher degree of inflammatory response in healed plaques. Moreover, the gender differences might account for the heterogeneity in macrophage accumulation.

In the ACS and SAP subgroup analysis related to microvessel, higher heterogeneity was found in the SAP group. The mean level of LDL-C was related to heterogeneity in microvessel among SAP patients in meta-regression. On the basis of the microvessel formation, inward growth is a common feature during thrombus organization, and it is more common in healed plaques (3). Importantly, the membranes of microvessels can be used as a source of free cholesterol in plaques, and in this way, the number of microvessels increases with the development of fatty plaques (4). The abovementioned mechanism might explain the relationship between mean LDL-C and microvessels in healed plaques. However, microvessels may also play a role at the cellular and molecular levels, which deserves further studies.

In the clinical therapeutic interventions, Kurihara et al. found that stents had limited dilation and more eccentric lumens in non-healed plaques after PCI in patients with ACS compared with healed plaques. Afterward, they found that the presence of healed plaques in culprit vessels was an independent predictor of revascularization rate during a 2-year follow-up (26, 27). In the early days, the complex characteristics of TCFA and microvessels as determined by OCT seemed to be potential predictors of subsequent progression of coronary plaque in patients with CAD (39). In a recent study by Prati et al., OCT-defined high-risk plaque based on MLA, thickness of fibrous cap, and content of macrophages were shown to be independent predictors at the 1-year follow-up (40). Similarly, the detection of healed plaques and their characteristics may help in identifying patients who are relatively immune to acute occlusion during periods of plaque instability, thereby further helping to stratify the risk and potentially contribute to treatment (41).

### Strengths and Limitations of This Study

Our study obtained novel results by including the recent studies and correct data. Besides, we conducted subgroup and meta-regression analyses to explore the relationship between the characteristics of healed plaque and important clinical variables; meanwhile, we have listed the OCT images of the healed plaque and atherosclerotic patterns in Supplementary Figure 6. However, there are some potential limitations to our analysis that should be considered when interpreting the findings. First, owing to the limited number of cohort or case–control studies on healed plaques and the possibility that certain unpublished articles and data might have been overlooked, 13 studies were finally included in this meta-analysis. Second, the OCT-related images in the included studies may have been affected by human judgment that introduced a degree of heterogeneity into the analysis. Third, owing to the inadequate numbers of trials, the funnel plots shown in this study might not precisely reflect the publication bias, which could be corrected by including additional randomized controlled trials in the future. Fourth, considering that plaque healing is regarded as a sign of past plaque instability, it might be that deep macrophage strakes or other variables caused an intraplaque change in signal intensity. Consistently, it is rather uneasy to address the presence of the microvessels. Finally, in addition to the possible sources of heterogeneity mentioned above, other factors such as medication could have also led to heterogeneity.

## Data Availability Statement

The original contributions presented in the study are included in the article/Supplementary Material, further inquiries can be directed to the corresponding author/s.

## Author Contributions

XF, QG, and YZ developed the initial idea of this study. XF and QG conducted a comprehensive search of three databases and took responsibility for selecting the study and extracting data. XF analyzed the data and drafted the article. QG and YZ carefully examined this manuscript. All authors agreed with the ideas presented in the article and have made their contributions to research design, interpretation of results, and ideas for writing articles.

## Funding

This study was supported by grants from National Key Research and Development Program of China (2017YFC0908800), Beijing Municipal Health Commission (Grant Nos. PXM2020_026272_000002 and PXM2020_026272_000014), and Natural Science Foundation of Beijing, China (Grant No. 7212027) to YZ. QG was supported by Natural Science Foundation of Beijing, China (Grant No. 7214223).

## Conflict of Interest

The authors declare that the research was conducted in the absence of any commercial or financial relationships that could be construed as a potential conflict of interest.

## Publisher's Note

All claims expressed in this article are solely those of the authors and do not necessarily represent those of their affiliated organizations, or those of the publisher, the editors and the reviewers. Any product that may be evaluated in this article, or claim that may be made by its manufacturer, is not guaranteed or endorsed by the publisher.

## Abbreviations

ACS: acute coronary syndrome
OCT: Optical coherence tomography
TCFA: thin cap fibroatheroma
CAD: coronary artery disease
PRISMA: Preferred Reporting Items for Systematic Reviews and Meta-Analyses
SAP: stable angina pectoris
SD: standard deviation
OR: odds ratio
CI: confidence interval
LDL-C: low-density lipoprotein cholesterol
MLA: minimal lumen area
RLA: reference lumen area
AS: area stenosis.
