# Supplementary material for: Prevalence of Healed Plaque and Factors Influencing Its Characteristics Under Optical Coherence Tomography in Patients With Coronary Artery Disease: A Systematic Review, Meta-Analysis, and Meta-Regression
Source: Front Cardiovasc Med. 2021 Nov 22;8:761208. doi: 10.3389/fcvm.2021.761208 (PMC8645588; doi:10.3389/fcvm.2021.761208)
Supplement: Supplementary file 1 [file Data_Sheet_1.docx]

**SUPPLEMENTAL MATERIAL**

**Prevalence of healed plaque and** **factors influencing its characteristics under optical coherence tomography in patients with coronary artery disease: a systematic review, meta-analysis and meta-regression**

Xunxun Feng, Yang Liu, Jiaqi Yang, Guangyao Zhai, Yujie Zhou* and Qianyun Guo*

**Table of Contents**

Table S1 Searching strategy for Medline .......................................................................3

Table S2 Searching strategy for Embase.........................................................................4

Table S3 Searching strategy for Cochrane .....................................................................5

Table S4 Assessment of study quality using the Newcastle-Ottawa scale ................... 6

Table S5 Specific definitions of each characteristic in healed plaque in every studies.7

Table S6 Egger test for prevalence of healed plaque.................................................. 12

Table S7 Publication bias detection using Egger test for every characteristic in healed plaque ..........................................................................................................................13

Figure S1 The flow diagram for selection of studies.................................................... 14

Figure S2 Forest plots of pooled odds ratio estimates stratified by culprit plaques vs. non-culprit plaques in qualitative characteristics of plaques........................................ 15

Figure S3 Forest plots of pooled weighted mean difference estimates stratified by culprit plaques vs. non-culprit plaques in quantitative characteristics of plaques..........16

Figure S4 Forest plots of pooled odds ratio estimates stratified by ACS vs. SAP in qualitative characteristics of plaques............................................................................17

Figure S5 Forest plots of pooled weighted mean difference estimates stratified by ACS vs. SAP in quantitative characteristics of plaques.........................................................18

Figure S6 OCT images of the healed plaque and atherosclerotic patterns………………………………………….........................................................19

Table S1 Searching strategy for Medline

Total number of results = 57

| # | Searches |  | Number of results |
| --- | --- | --- | --- |
| 1 | Plaque, Atherosclerotic | [Mesh] |  |
| 2 | Plaques, Atherosclerotic | [Title/Abstract] |  |
| 3 | Atherosclerotic Plaques | [Title/Abstract] |  |
| 4 | Atherosclerotic Plaque | [Title/Abstract] |  |
| 5 | Fatty Streak, Arterial | [Title/Abstract] |  |
| 6 | arterial fatty streaks | [Title/Abstract] |  |
| 7 | Arterial Fatty Streaks | [Title/Abstract] |  |
| 8 | Fatty Streaks, Arterial | [Title/Abstract] |  |
| 9 | Streak, Arterial Fatty | [Title/Abstract] |  |
| 10 | Streaks, Arterial Fatty | [Title/Abstract] |  |
| 11 | fibro atheroma | [Title/Abstract] |  |
| 12 | fibro atheromas | [Title/Abstract] |  |
| 13 | fibro atheromatous plaques | [Title/Abstract] |  |
| 14 | fibro atheromatous plaque | [Title/Abstract] |  |
| 15 | Plaque, fibro atheromatous | [Title/Abstract] |  |
| 16 | Plaques, fibro atheromatous | [Title/Abstract] |  |
| 17 | Atheroma | [Title/Abstract] |  |
| 18 | Atheromas | [Title/Abstract] |  |
| 19 | Atheromatous Plaques | [Title/Abstract] |  |
| 20 | Plaque, Atheromatous | [Title/Abstract] |  |
| 21 | Plaques, Atheromatous | [Title/Abstract] |  |
| 22 | 1 or 2 or 3 or 4 or 5 or 6 or 7 or 8 or 9 or 10 or 11 or 12 or 13 or 14 or 15 or 16 or 17 or 18 or 19 or 20 or 21 | | 29484 |
| 23 | Tomography, Optical Coherence | [Mesh] |  |
| 24 | Coherence Tomography, optical | [Title/Abstract] |  |
| 25 | OCT Tomography | [Title/Abstract] |  |
| 26 | Tomography, OCT | [Title/Abstract] |  |
| 27 | Optical Coherence Tomography | [Title/Abstract] |  |
| 28 | 23 or 24 or 25 or 26 or 27 | | 54126 |
| 29 | Wound Healing | [Mesh] |  |
| 30 | Healing | [Title/Abstract] |  |
| 31 | Healings | [Title/Abstract] |  |
| 32 | Healed | [Title/Abstract] |  |
| 33 | Layered | [Title/Abstract] |  |
| 34 | 29 or 30 or 31 or 32 or 33 | | 312959 |
| 35 | 22 and 28 and 34 | | 57 |

Table S2. Searching strategy for Embase

Total number of results = 119

| # | Searches | Number of results |
| --- | --- | --- |
| 1 | 'atherosclerotic plaque'/exp | 35496 |
| 2 | 'plaques, atherosclerotic':ti,ab or 'atherosclerotic plaques':ti,ab or 'atherosclerotic plaque':ti,ab or 'fatty streak, arterial':ti,ab or 'arterial fatty streak':ti,ab or 'arterial fatty streaks':ti,ab or 'fatty streaks, arterial':ti,ab or 'streak, arterial fatty':ti,ab or 'streaks, arterial fatty':ti,ab or 'fibroatheroma':ti,ab or 'fibroatheromas':ti,ab or 'fibroatheromatous plaques':ti,ab or 'fibroatheromatous plaque':ti,ab or 'plaque, fibroatheromatous':ti,ab or 'plaques, fibroatheromatous':ti,ab or 'atheromas':ti,ab or 'atheromas':ti,ab or 'atheromatous plaques':ti,ab or 'atheromatous plaque':ti,ab or 'plaques, atheromatous':ti,ab or 'plaques, atheromatous':ti,ab | 30195 |
| 3 | 1 OR 2 | 46939 |
| 4 | 'optical coherence tomography'/exp | 73942 |
| 5 | 'coherence tomography, optical':ti,ab or 'oct tomography':ti,ab or 'tomography, oct':ti,ab or 'optical coherence tomography':ti,ab | 56066 |
| 6 | 4 OR 5 | 77719 |
| 7 | 'healing'/exp | 274766 |
| 8 | 'healing':ti,ab or 'healings':ti,ab or 'healed':ti,ab or 'layered':ti,ab | 303768 |
| 9 | 7 OR 8 | 437099 |
| 10 | 3 AND 6 AND 9 | 119 |

Table S3. Searching strategy for Cochrane

Total number of results = 7

| # | Searches | Number of results |
| --- | --- | --- |
| 1 | MeSH descriptor: [Plaque, Atherosclerotic] explode all trees | 295 |
| 2 | (Plaques, Atherosclerotic):ti,ab,kw OR (Atherosclerotic Plaques):ti,ab,kw OR (Atherosclerotic Plaque):ti,ab,kw OR (Fatty Streak, Arterial):ti,ab,kw OR (Arterial Fatty Streak):ti,ab,kw OR (Arterial Fatty Streaks):ti,ab,kw OR (Fatty Streaks, Arterial):ti,ab,kw OR (Streak, Arterial Fatty):ti,ab,kw OR (Streaks, Arterial Fatty):ti,ab,kw OR (Fibroatheroma):ti,ab,kw OR (Fibroatheromas):ti,ab,kw OR (Fibroatheromatous Plaques):ti,ab,kw OR (Fibroatheromatous Plaque):ti,ab,kw OR (Plaque, Fibroatheromatous):ti,ab,kw OR (Plaques, Fibroatheromatous):ti,ab,kw OR (Atheroma):ti,ab,kw OR (Atheromas):ti,ab,kw OR (Atheromatous Plaques):ti,ab,kw OR (Atheromatous Plaque):ti,ab,kw OR (Plaque, Atheromatous):ti,ab,kw OR (Plaques, Atheromatous):ti,ab,kw | 1828 |
| 3 | 1 OR 2 | 1828 |
| 4 | MeSH descriptor: [Tomography, Optical Coherence] explode all trees | 1460 |
| 5 | (Coherence Tomography, Optical):ti,ab,kw OR (OCT Tomography):ti,ab,kw OR (Tomography, OCT):ti,ab,kw OR (Optical Coherence Tomography):ti,ab,kw | 4332 |
| 6 | 4 OR 5 | 4332 |
| 7 | MeSH descriptor: [Wound Healing] explode all trees | 6093 |
| 8 | (healing):ti,ab,kw OR (healings):ti,ab,kw OR (healed):ti,ab,kw OR (layered):ti,ab,kw | 26991 |
| 9 | 7 OR 8 | 27615 |
| 10 | 3 AND 6 AND 9 | 7 |

Table S4 Assessment of study quality using the Newcastle-Ottawa scale

| Author | Publication  year | selection | Comparability | Outcome/exposure | Total  score |
| --- | --- | --- | --- | --- | --- |
| Shimokado et al | 2018 | *** | - | ** | 5 |
| Fracassi et al | 2019 | **** | - | *** | 7 |
| Okamoto et al | 2019 | *** | - | ** | 5 |
| Russo et al | 2019 | **** | - | ** | 6 |
| Wang et al | 2019 | **** | - | *** | 7 |
| Araki et al | 2020 | *** | - | ** | 5 |
| Kurihara et al-1 | 2020 | *** | - | *** | 6 |
| Kurihara et al-2 | 2020 | *** | - | *** | 6 |
| Russo et al | 2020 | **** | - | ** | 6 |
| Usui et al | 2020 | *** | - | *** | 6 |
| Dai et al | 2020 | **** | - | *** | 7 |
| Li et al | 2021 | *** | - | ** | 6 |
| Kimura et al | 2021 | *** | - | ** | 6 |

Table S5 Specific definitions of each characteristic in healed plaque in every studies

|  | TCFA | Plaque rupture | Macrophage accumulation | Microvessel | Thrombus | Calcification | MLA | RLA | AS | Lesion length |
| --- | --- | --- | --- | --- | --- | --- | --- | --- | --- | --- |
| Shimokado et al, 2018 | not mentioned | a necrotic core in continuity with the superimposed fibrous tissue layer with a discrete defect in the fibrous cap | signal-rich, distinct or confluent punctuate regions with shadowing | no-signal tubuloluminal structures without a connection to the vessel lumen recognized on 3 consecutive cross-sectional OCT images | not mentioned | | automated lumen contour detection followed by additional manual correction | | | |
| Fracassi et al, 2019 | a plaque with the thinnest part of the fibrous cap measuring <65μm and with a lipid arc wider than 90° | presence of fibrous cap discontinuity with cavity formation | presence of highly backscattering focal granular regions in the fibrous cap | signal-poor vesicular or tubular structure delineated in multiple contiguous frames | an irregular mass floating in or protruding into the lumen with a dimension of at least 250 μm | an area with low backscattering signal and a sharp border inside of a plaque | the smallest lumen area within the length of the lesion | the mean of the largest lumen area proximal and distal to the stenosis | the percentage decrease of lumen area at the narrowest frame | quantitative coronary angiography analysis program (CAAS 5.10.1, Pie Medical Imaging BV, Maastricht, the Netherlands) |
| Okamoto et al, 2019 | a large lipid pool (≥1 quadrants) covered with a thin fibrous cap (cap thickness, 65 μm) | a disrupted fibrous membrane with an underlying empty cavity | strong, linear images on the plaque surface accompanied by high attenuation | no-signal tubuloluminal structures without a connection to the vessel lumen seen on 3 consecutive cross-sectional OCT images | a sample that was rich in platelets, fibrin, or erythrocytes in a continuous histopathological section | heterogeneous, sharply delineated, signal-poor or signal-rich regions or alternating signal poor and signal-rich regions | not mentioned | | | |
| Russo et al, 2019 | not mentioned | | | | | | quantitative coronary angiogram (QCA) analysis software (CAAS 5.10.1, Pie Medical Imaging BV,  Maastricht, The Netherlands) | | | |
| Wang et al, 2019 | a plaque with lipid content in at least two quadrants, with the thinnest part of the fibrous cap measuring less than 65 μm | not mentioned | not mentioned | Not mentioned | a mass floating in or protruding into the lumen with a dimension of at least 250 μm | not mentioned | quantitative coronary angiogram analysis program (CAAS 5.10.1; Pie Medical Imaging BV, Maastricht, the Netherlands) | | | |
| Araki et al, 2020 | a plaque with the thinnest part of the fibrous cap measuring < 65 μm and with a lipid arc > 90° | not mentioned | not mentioned | not mentioned | not mentioned | not mentioned | quantitative coronary angiography (QCA) software (CASS 5.10.1, Pie Medical Imaging BV, Maastricht, The Netherlands) | | | |
| Kurihara et al-1, 2020 | post-procedure qualitative and quantitative assessments were evaluated using the previously validated criteria (using references not mentioned in details) | | | | | | | | | |
| Kurihara et al-2, 2020 | not mentioned | | | | | | | | | |
| Russo et al, 2020 | not mentioned | not mentioned | the presence of superficial highly backscattering focal granular regions | signal-poor vesicular or tubular structure delineated in at least 3 contiguous frames | an irregular mass attached to the luminal surface or floating into the lumen with diameter >250 µm | lesions with maximal arc ≥90° and length ≥of 4 mm | the smallest lumen area found within the length of the entire lesion | the mean of the largest lumen area proximal and distal to the stenosis within 10 mm from the edge of the plaque | [mean reference lumen area−MLA]/mean reference lumen area×100 | quantitative coronary angiography analysis software (CASS 5.10.1, Pie Medical Imaging BV, Maastricht, the Netherlands) |
| Usui et al, 2020 | a fibrous cap thickness < 65 μm with lipid-rich plaque | not mentioned | signal-rich, distinct, or confluent punctate region | non-signal tubuloluminal structures without a connection to the vessel lumen | not mentioned | calcified plaque as a signal-poor or heterogeneous region with a sharply delineated border; spotty calcification as a small calcium deposit with an angle < 90° | per lesion measured | not mentioned | | |
| Dai et al, 2020 | qualitative and quantitative analyses of plaque characteristics, as well as inter- and intraobserver agreement have been detailed in Methods in the Data Supplement (not obtained) | | | | | | | | | |
| Li et al, 2021 | not mentioned | disrupted fibrous cap with a clear cavity formation | not mentioned | not mentioned | not mentioned | not mentioned | not mentioned | | | |
| Kimura et al, 2021 | lipid-rich plaques with a fibrous cap thickness < 70 μm | a fibrous cap discontinuity with cavity formation | signal-rich, distinct or confluent punctuate regions with shadowing | no-signal tubuloluminal structures without a connection to the vessel lumen seen on 3 consecutive cross-sectional OCT images | a mass attached to luminal surface or floating within the lumen | a signal-poor or heterogeneous region with a sharply delineated border | quantitative coronary angiography was performed using CAAS 4.1.1 software (Pie Medical Imaging, Maastricht, The Netherlands) | | | |

Table S6 Egger test for prevalence of healed plaque

| Groups | P |
| --- | --- |
| Overall population | 0.947 |
| ACS | 0.836 |
| SAP | 0.907 |
| Culprit Plaque | 0.689 |
| Non-culprit Plaque | - (studies less than 5) |

Table S7 Publication bias detection using Egger test for every characteristic in healed plaque

| Characteristics | Coef (95% CI) | P |
| --- | --- | --- |
| TCFA | 0.82 (-1.84-3.48) | 0.497 |
| Plaque rupture | 1.93 (-1.68-5.53) | 0.228 |
| Macrophage accumulation | 1.66 (-1.60-4.92) | 0.282 |
| Microvessel | 0.82 (-1.98-3.63) | 0.524 |
| Thrombus | -0.40 (-4.79-3.99) | 0.813 |
| Calcification | -0.94 (-2.75-0.86) | 0.271 |
| MLA | 3.70 (-1.69-9.10) | 0.159 |
| RLA | 3.52 (-0.37-7.40) | 0.070 |
| AS | -2.50 (-8.28-3.29) | 0.349 |
| Lesion length | -3.20 (-7.43-1.04) | 0.096 |

Figure S1 The flow diagram for selection of studies


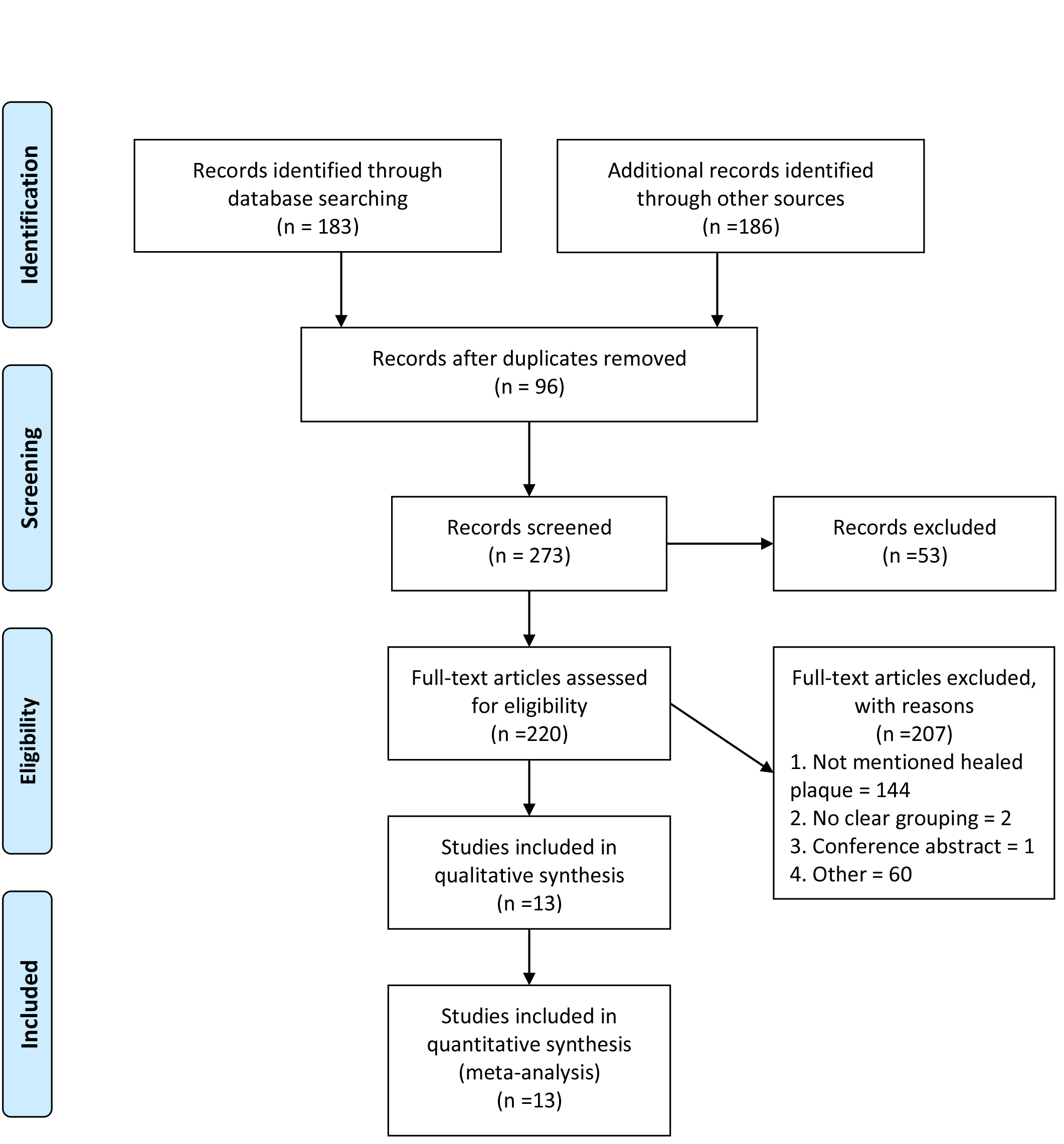


Figure S2 Forest plots of pooled odds ratio estimates stratified by culprit plaques vs. non-culprit plaques in qualitative characteristics of plaques


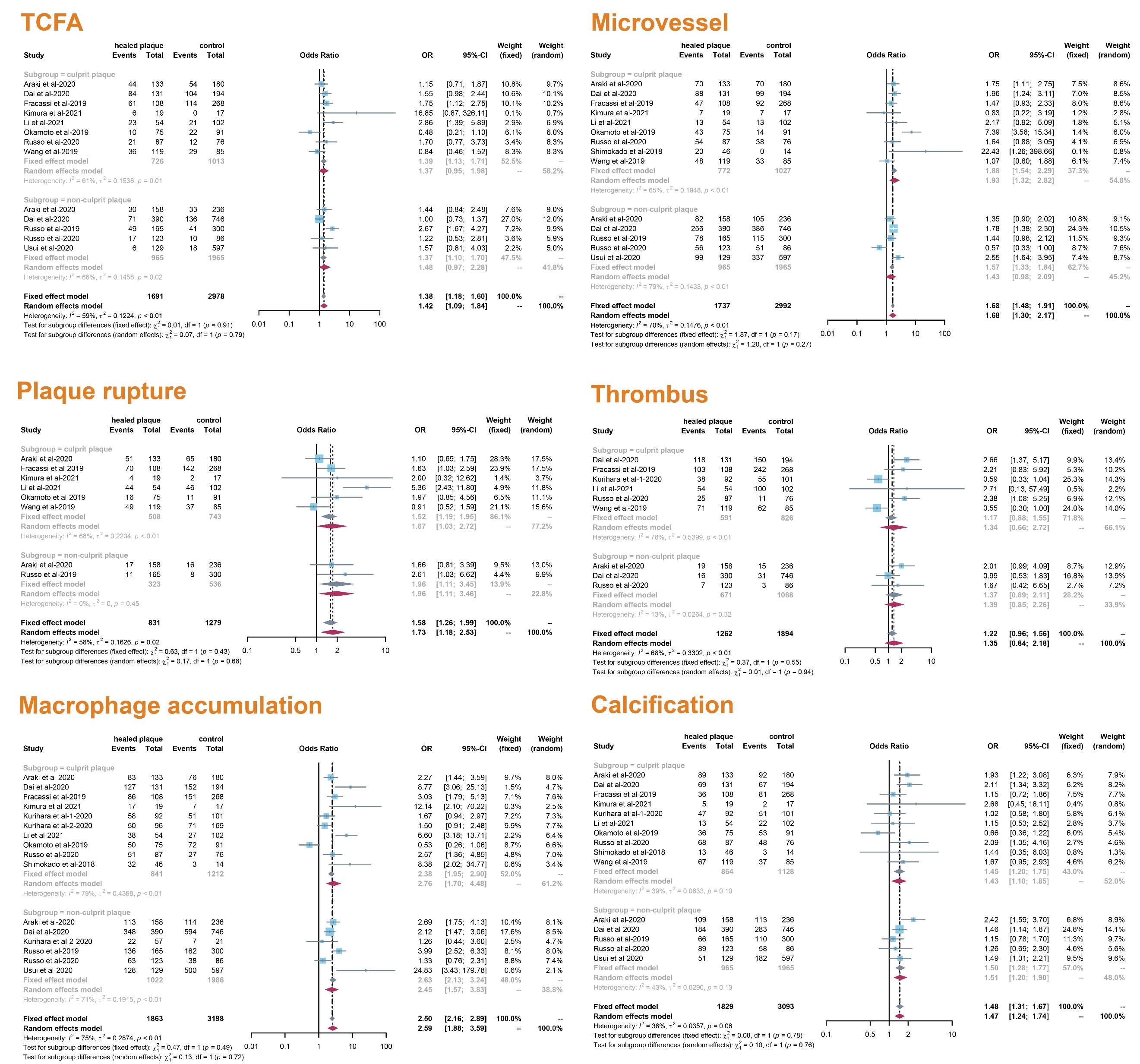


Figure S3 Forest plots of pooled weighted mean difference estimates stratified by culprit plaques vs. non-culprit plaques in quantitative characteristics of plaques


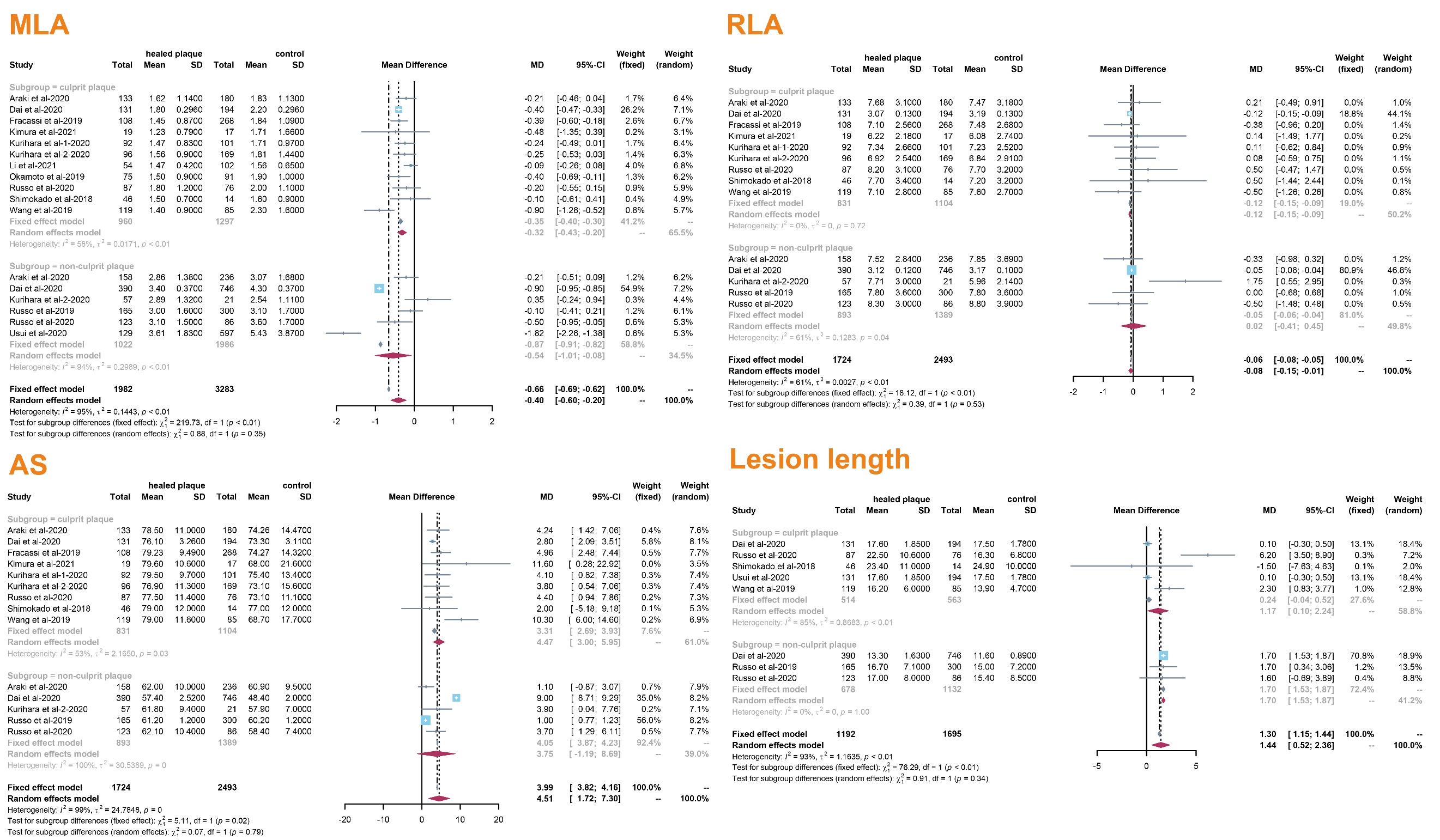


Figure S4 Forest plots of pooled odds ratio estimates stratified by ACS vs. SAP in qualitative characteristics of plaques


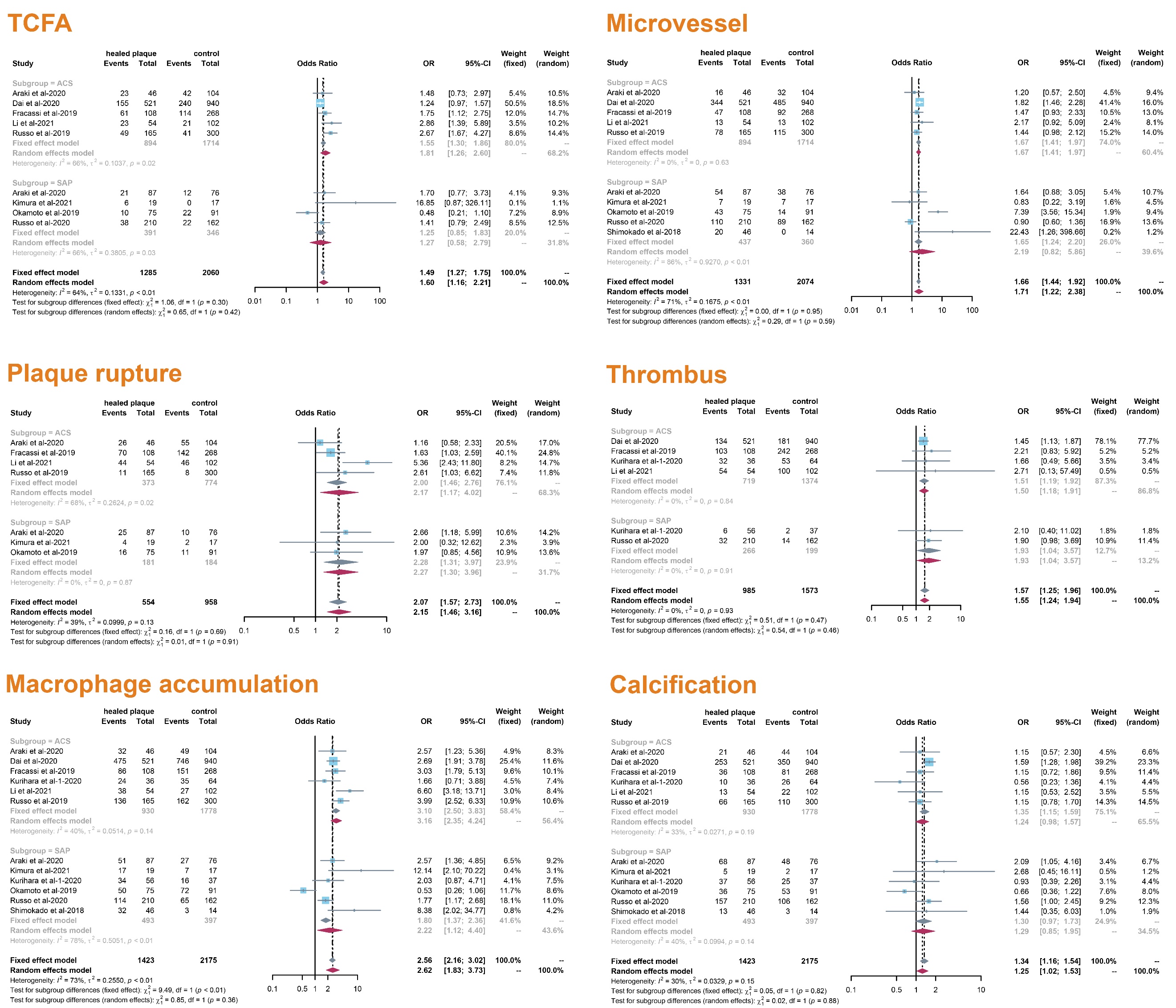


Figure S5 Forest plots of pooled weighted mean difference estimates stratified by ACS vs. SAP in quantitative characteristics of plaques


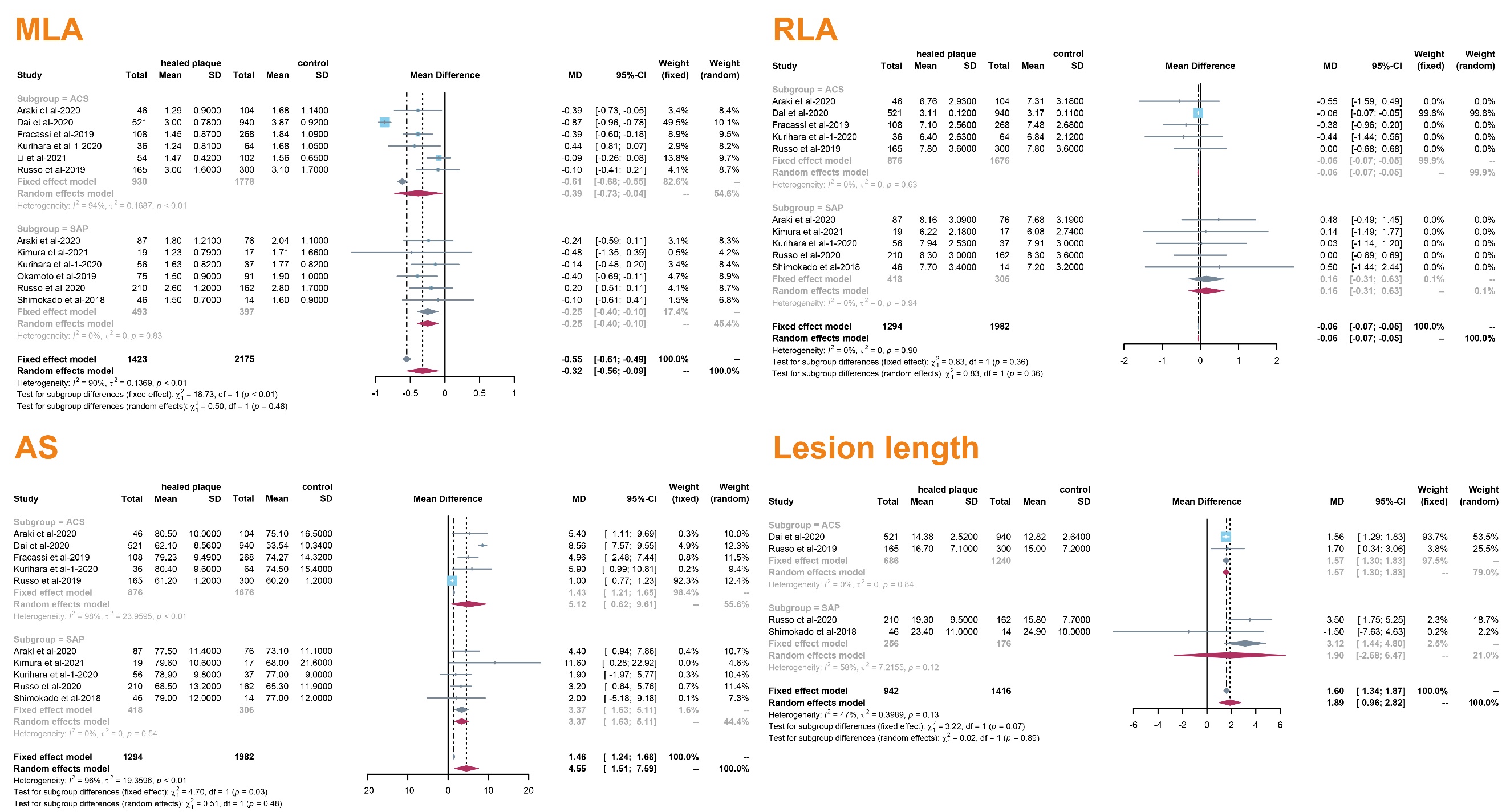


Figure S6 OCT images of the healed plaque and atherosclerotic patterns


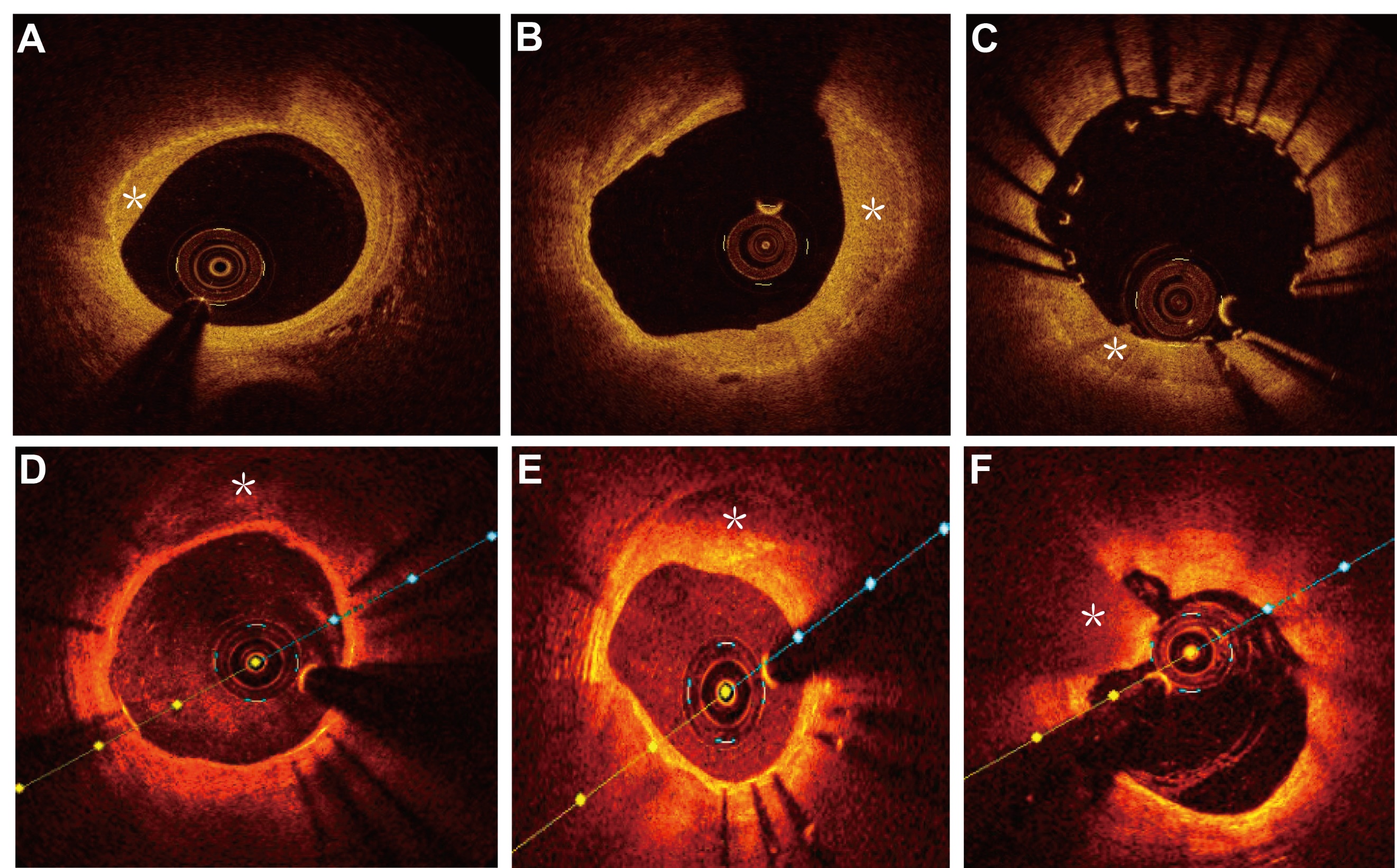


Asterisks in (A), (B), and (C) represent the healed plaque. The asterisk in (D) represent the calcification site. The asterisk in (E) represent the fatty plaque. The asterisk in (F) represent the red thrombus. (Images were reviewed and approved by the Institutional Ethics Committee of Beijing Anzhen Hospital.)
